# Non-invasive assessment of cerebral oxygenation: A comparison of retinal and transcranial oximetry

**DOI:** 10.1371/journal.pone.0190612

**Published:** 2018-01-05

**Authors:** Karel Van Keer, Jan Van Keer, João Barbosa Breda, Vahid Nassiri, Cathy De Deyne, Cornelia Genbrugge, Luís Abegão Pinto, Ingeborg Stalmans, Evelien Vandewalle

**Affiliations:** 1 Department of Ophthalmology, University Hospitals Leuven, Leuven, Belgium; 2 Department of Cardiology, University Hospitals Leuven, Leuven, Belgium; 3 I-BioStat, KU Leuven, Leuven, Belgium; 4 Faculty of Medicine and Life Sciences, Hasselt University, Hasselt, Belgium; 5 Department of Anesthesiology, Intensive Care, Emergency Medicine and Pain Therapy, Ziekenhuis Oost-Limburg, Genk, Belgium; 6 Department of Ophthalmology, Centro Hospitalar Lisboa Norte, Lisbon, Portugal; 7 Department of Ophthalmology Neurosciences, Laboratory of Ophthalmology, KU Leuven, Leuven, Belgium; Tokai University, JAPAN

## Abstract

**Background:**

To investigate the correlation between cerebral (SO_2-transcranial_), retinal arterial (SaO_2-retinal_) and venous (SvO_2-retinal_) oxygen saturation as measured by near-infrared spectroscopy (NIRS) and retinal oximetry respectively.

**Methods:**

Paired retinal and cerebral oxygen saturation measurements were performed in healthy volunteers. Arterial and venous retinal oxygen saturation and diameter were measured using a non-invasive spectrophotometric retinal oximeter. Cerebral oxygen saturation was measured using near-infrared spectroscopy. Correlations between SO_2-transcranial_ and retinal oxygen saturation and diameter measurements were assessed using Pearson correlation coefficients. Lin’s concordance correlation coefficient (CCC) and Bland-Altman analysis were performed to evaluate the agreement between SO_2-transcranial_ as measured by NIRS and as estimated using a fixed arterial:venous ratio as 0.3 x SaO_2-retinal_ + 0.7 x SvO_2-retinal_. The individual relative weight of SaO_2-retinal_ and SvO_2-retinal_ to obtain the measured SO_2-transcranial_ was calculated for all subjects.

**Results:**

Twenty-one healthy individuals aged 26.4 ± 2.2 years were analyzed. SO_2-transcranial_ was positively correlated with both SaO_2-retinal_ and SvO_2-retinal_ (r = 0.44, p = 0.045 and r = 0.43, p = 0.049 respectively) and negatively correlated with retinal venous diameter (r = -0.51, p = 0.017). Estimated SO_2-transcranial_ based on retinal oximetry showed a tolerance interval of (-13.70 to 14.72) and CCC of 0.46 (95% confidence interval: 0.05 to 0.73) with measured SO_2-transcranial_. The average relative weights of SaO_2-retinal_ and SvO_2-retinal_ to obtain SO_2-transcranial_ were 0.31 ± 0.11 and 0.69 ± 0.11, respectively.

**Conclusion:**

This is the first study to show the correlation between retinal and cerebral oxygen saturation, measured by NIRS and retinal oximetry. The average relative weight of arterial and venous retinal oxygen saturation to obtain the measured transcranial oxygen saturation as measured by NIRS, approximates the established arterial:venous ratio of 30:70 closely, but shows substantial inter-individual variation. These findings provide a proof of concept for the role of retinal oximetry in evaluating cerebral oxygenation.

## Introduction

The brain is highly dependent on aerobic metabolism to meet its energy demand. [1] Prolonged ischemia leads to neuronal injury and is associated with poor neurological outcome. [2–7] Because severe cerebral ischemia may exist in the absence of systemic hypoxia, there is a need for selective assessment of the central nervous system oxygenation. [8,9]

Transcranial near-infrared spectroscopy (NIRS) is increasingly used in clinical practice to measure cerebral saturation non-invasively. [10] NIRS relies on the differences in the absorption spectra of a near-infrared light beam between oxygenated and de-oxygenated hemoglobin. [11] NIRS has proven useful in a variety of clinical settings, especially during cardiac surgery. [4,12,13] However, current NIRS devices have several drawbacks. First, despite the use of different source-detector distances, NIRS is not entirely selective for the cerebral saturation: the measured oxygen saturation is "contaminated" by the saturation of the more superficial dura, skull and skin. [14,15] Second, the saturation measured by NIRS is in fact a composite of arterial, capillary and venous blood. [16] For the FORE-SIGHT^®^ transcranial oximeter (CAS Medical Systems, Branford, CT, USA) the relative contribution of arterial and venous blood (arterial:venous ratio) to the measured composite value is 30:70, but the inter-individual variability of this ratio is considerable. [16–20] This is especially relevant in critically ill patients in whom altered perfusion pressures, disturbed (auto)regulation and administration of vasoactive drugs are likely to influence this ratio. [21–23]

**Fig 1:**
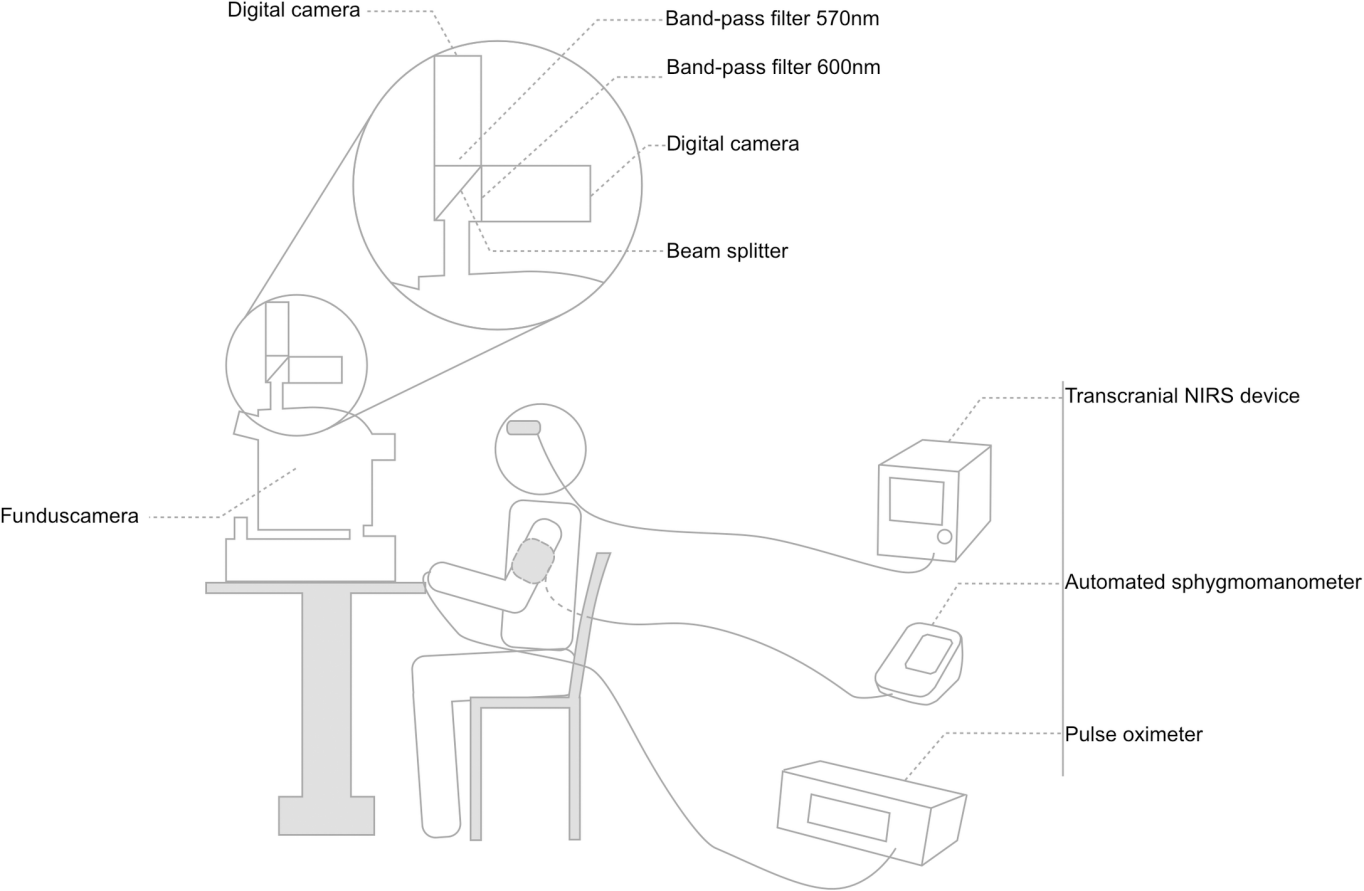
Experimental setup. Experimental setup showing seated subject, retinal oximeter, NIRS device, sphygmomanometer and pulse oximeter. Magnified detail of the dual-wavelength retinal oximeter consisting of a funduscamera, beam splitter, two band-pass filters and two digital cameras.

**Fig 2:**
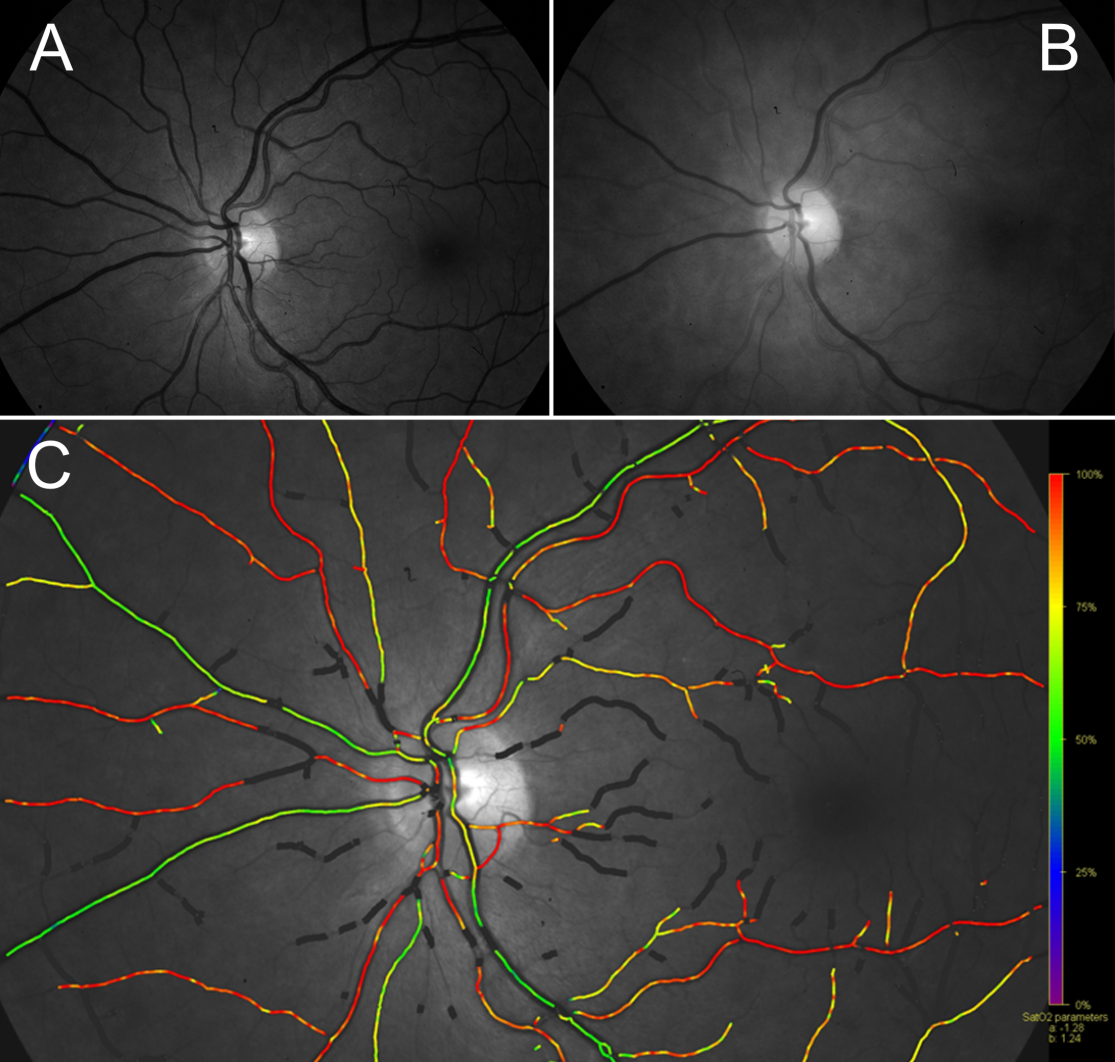
Retinal oximetry. Sample monochromatic retinal image at 570nm (A) and 600nm (B) and resulting pseudo-color oxygen saturation overlay map (C), showing oxygen saturation in arteries (red) and veins (green) separately. Note the darker appearance of veins compared to arteries at 600nm (B) but not at 570nm (A).

These shortcomings could partially be addressed by looking at the central nervous system oxygen saturation from a different point of view. Thanks to the transparency of the ocular media, the human eye offers a convenient way to study the circulatory system. In recent years, several new diagnostic tools have been developed to investigate the retinal circulation. Dual-wavelength spectrophotometric retinal oximetry is an imaging technique that allows the visualization and measurement oxygen saturation in retinal blood vessels by comparing the relative reflectance of retinal blood vessels at 570nm and 600nm. [24] At 570nm light, oxidized and deoxidized hemoglobin absorb light equally (isosbestic wavelength), while at 600 nm deoxidized hemoglobin absorbs more light relative to oxidized hemoglobin (non-isosbestic wavelength). [25] (Fig 1). The retinal oximeter simultaneously acquires monochromatic retinal fundus pictures at 570nm and 600nm using a dual camera setup coupled to a conventional fundus camera. Upon acquisition, the unmodified xenon flash from the fundus camera illuminates the retina. The reflected light is focused in the funduscamera. Unlike in a conventional funduscamera, the image is not directly captured by a single digital camera. Rather, the optical pathway is split by a beam splitter, filtered by two narrow band-pass filters of respectively 570nm and 600nm and subsequently registered on two different digital cameras. [24] (Figs 1 and 2) By analyzing the ratio of the optical density of retinal blood vessels at both wavelengths, oxygen saturation inside the retinal blood vessels can be calculated based on the nearly linear inverse relationship between optical density ratio and hemoglobin oxygen saturation. [25] A more in-depth theoretical explanation has been published elsewhere. [26] The oxygen saturation inside the retinal vessels is displayed as a pseudo-color overlay on top of the retinal fundus picture and is measured manually by selecting a retinal blood vessel. Vessels can then be labeled as either arteries or veins based on saturation and continuity with feeder vessels. (Fig 2) Hence, in contrast to NIRS, retinal oximetry does provide a way to measure arterial and venous oxygen saturations separately at a high spatial resolution of less than 10μm. [24,27,28] Using the unmodified xenon flash of a conventional funduscamera, which is safe for human use according to American National Standards Institute (ANSI) standards, and requiring only pupil dilation, retinal oximetry provides a safe and convenient imaging modality. [29,30] Over the last decade, retinal oximetry has become an established tool for the assessment of oxygen saturation in different ocular diseases. [31–36]However, the impact of systemic parameters on retinal vessel oxygen saturation has been investigated less extensively so far. [37–41] As the retinal and cerebral circulation share a common embryological, anatomical and physiological background, retinal oximetry may also be a valuable tool in assessing cerebral oxygenation. [42–45]

This pilot study aims to provide a proof of concept for the use of retinal oximetry in assessing cerebral oxygen saturation by investigating the correlation between retinal arterial (SaO_2-retinal_) and venous oxygen saturation (SvO_2-retinal_) measured by spectrophotometric retinal oximetry and cerebral oxygen saturation (SO_2-transcranial_) measured by NIRS.

## Methods

### Study population

Healthy adult volunteers were recruited at the Department of Ophthalmology of the University Hospitals Leuven. Subjects taking vasoactive medication or with a history of ocular, vascular or neurological diseases were excluded. A written informed consent in accordance with the tenets of the Declaration of Helsinki was obtained from each participant prior to any examinations. The study was approved by the local ethics committee (Medical Ethical Board of the University Hospitals Leuven, Belgium ML9229).

### Experimental design

Subjects were asked to refrain from caffeine, nicotine and physical exercise for 12h before testing. After instillation of phenylephrine 5% (Neosynephrine-POS, Ursapharm, Saarbrucken, Germany) and Tropicamide 0,5% (Tropicol, Théa Pharma, Wetteren, Belgium) in the left eye, a standard protocol, that has shown to result in good pupil dilation (required for the retinal oximeter that was used in this study) without influencing retinal oxygen saturation values, was performed. [46] Participants were seated in an upright position for at least 20 minutes prior to image acquisition. A pulse oximeter was placed on the left index finger (Ohmeda TruSat Pulse Oximeter, GE Healthcare, Finland) and an automated sphygmomanometer was placed on the right upper arm (Omron HEM-7001-E, Omron, Kyoto, Japan). The NIRS probe was placed on the left side of the forehead below the hairline facing the left frontal lobe (FORE-SIGHT^®^ technology, CAS Medical Systems, Branford, CT, USA). Heart rate, pulse oxygen saturation (SO_2-pulse_), systolic and diastolic blood pressure were measured. SO_2-transcranial_ was recorded once the pupil was dilated to a diameter of at least 5mm and continuously monitored during 10 minutes. Following two minutes of dark-adaptation, retinal fundus images were acquired using a Oxymap^®^ T1 oximeter (Oxymap ehf., Reykjavik, Iceland). The experimental setup is summarized in Fig 1.

The quality of the oximetry images was evaluated using a standardized protocol. Images were subjectively rated on focus, contrast, glare and shadow using a pass/fail system. Failure to pass any of these criteria led to exclusion of the image. [46] Image analysis was performed with the Oxymap^®^ Analyzer software version 2.4.0 using the following protocol: first-degree retinal vessels with a width of more than six pixels and a length between 50 and 200 pixels were selected from the color-coded saturation map. Second-degree vessels were only analyzed if first-degree vessels did not meet the criteria. Around the optic disc, an area of 15 pixels as well as branching vessels and their origin were manually excluded. Vessels were manually categorized as arteries or veins and subsequently averaged over the four quadrants. Vessel diameters were measured automatically using the Oxymap^®^ Analyzer software. [36]

Estimated SO_2-transcranial_ was calculated using the established relative arterial and venous weight of the FORE-SIGHT^®^ transcranial oximeter of 30:70 using Eq 1. [18]

$$0.3\times {SaO}_{2-retinal}+0.7\times {SvO}_{2-retinal}=estimated\;{SO}_{2-transcranial}$$ (1)

Individual arterial (w_*i*_) and venous (1 − w_*i*_) relative weight to obtain measured SO_2-transcranial_ was calculated in all subjects using Eq 2.

forparticipanti:

$${SO}_{2-{transcranial}_{i}}=w_{i}\times {SaO}_{2-{retinal}_{i}}+(1-w_{i})\times {SvO}_{2-{retinal}_{i}}$$ (2)

where

$$0\leq w_{i}\leq 1$$

For example, in participant *i* with a SaO_2-retinal_ of 95%, a SvO_2-retinal_ of 60% and a SO_2-transcranial_ of 70%, the arterial (w_*i*_) and venous weight (1 − w_*i*_) would be 0.29 and 0.71 respectively, since 0.29 x 95% + 0.71 x 60% = 70%.

The arterial and venous oxygen saturation contributing to the measured SO_2-transcranial_ were estimated by substituting SO_2-pulse_ for arterial and by estimating venous oxygen saturation (estimated SvO2_-transcranial_) using Eq 3.

$$\frac{{SO}_{2-transcranial}-0.3\times {SpO}_{2-pulse}}{0.7}=estimated\;{SvO}_{2-transcranial}$$ (3)

### Statistical analysis

Statistical analysis was performed with R (R Statistical Software version 3.4.1; R Foundation for Statistical Computing, Vienna, Austria) using the “BlandAltmanLeh”, “epiR” and “Tolerance” package. [47–49] Normality of continuous variables was tested using the Shapiro-Wilk test. Demographics and measurements were analyzed with descriptive statistics (mean ± standard deviation). Correlations were evaluated using Pearson correlation coefficients. The agreement between measured SO_2-transcranial_ and estimated SO_2-transcranial_ was evaluated using Lin’s concordance correlation coefficient (CCC) and Bland-Altman analysis. [50,51] In terms of bias, mean of the difference between SO_2-transcranial_ and estimated SO_2-transcranial_, its 95% confidence intervals and tolerance intervals (for 99% of future measurements to be within 95% confidence) were calculated. Statistical significance was based on two-sided p-values of <0.05. As this study was set up as a pilot study, power analysis and sample-size calculations were not performed. Individual data points and the R script that was used to generate the statistical analyses and figures can were uploaded as supporting information. (S1 File, S2 File)

## Results

A total of 22 healthy individuals, 6 men and 16 women, were initially recruited. One subject was excluded because of inadequate quality of the oximetry images. Descriptive statistics of demographic characteristics of the included subjects are summarized in Table 1.

**Table 1:** Characteristics of the study population*.

Age (years)		
	Mean	26.4 ± 2.2
	Range	[24–33]
Sex (F/M)		16/5
Systolic blood pressure (mmHg)		126.3 ± 10.3
Diastolic blood pressure (mmHg)		79.6 ± 8.0
Mean arterial pressure (mmHg)		95.1 ± 7.3
Heart rate (bpm)		72.1 ± 12.7

* *n* = 21 individuals

Data are presented as mean ± standard deviation. F, female; M, male; bpm, beats per minute

Average SO_2-pulse_, SaO_2-retinal_, SvO_2-retinal_ and SO_2-transcranial_ were 98.2 ± 1.1%, 95.3 ± 2.1%, 61.6 ± 4.0% and 72.2 ± 3.5%, respectively. SO_2-transcranial_ showed a significant positive correlation with both SaO_2-retinal_ and SvO_2-retinal_ (r = 0.44, p *=* 0.045 and r = 0.43, p *=* 0.049, respectively) and a negative correlation with venous diameter (r = -0.51, p *=* 0.017). (Table 2) Correlations between retinal, peripheral and transcranial saturations per vessel type are shown in Table 3.

**Table 2:** Correlation with transcranial saturation.

			Cerebral saturation	
			r	p
Peripheral				
	SO_2-pulse_ (%)	98.2 ± 1.1	-0.189	0.413
Retinal				
	SaO_2-retinal_ (%)	95.3 ± 2.1	**0.442**	**0.045**
	SvO_2-retinal_ (%)	61.6 ± 4.0	**0.434**	**0.049**
	Da_-retinal_ (μm)	110.4 ± 10.7	-0.121	0.601
	Dv_-retinal_ (μm)	140.9 ± 13.4	**-0.513**	**0.017**
Cerebral				
	SO_2-transcranial_ (%)	72.2 ± 3.5	1	/

Bold values represent significance at p <0.05. SO_2-pulse_, peripheral oxygen saturation; SaO_2-retinal_, mean retinal arterial oxygen saturation; SvO_2-retinal_, mean retinal venous oxygen saturation; SO_2-transcranial_, cerebral oxygen saturation; Da_-retinal_, mean retinal arterial diameter; Dv_-retinal_, mean retinal venous diameter

**Table 3:** Correlation coefficients per vessel type.

					r	p
arterial	SaO_2-retinal_ (%)	95.3 ± 2.1	SO_2-pulse_ (%)	98.2 ± 1.1	-0.103	0.658
venous	SvO_2-retinal_ (%)	61.6 ± 4.0	Estimated SvO_2-transcranial_ (%)	61.6 ± 5.1	0.422	0.056
mixed	Estimated SO_2-transcranial_ (%)	71.7 ± 3.2	SO_2-transcranial_ (%)	72.2 ± 3.5	0.467	0.032

SaO_2-retinal_, mean retinal arterial oxygen saturation; SvO_2-retinal_, mean retinal venous oxygen saturation; Estimated SO_2-transcranial_, estimated cerebral oxygen saturation (Eq 1); SO_2-pulse_, peripheral oxygen saturation; Estimated SO_2-transcranial_, estimated venous cerebral oxygen saturation (Eq 3)

**Fig 3:**
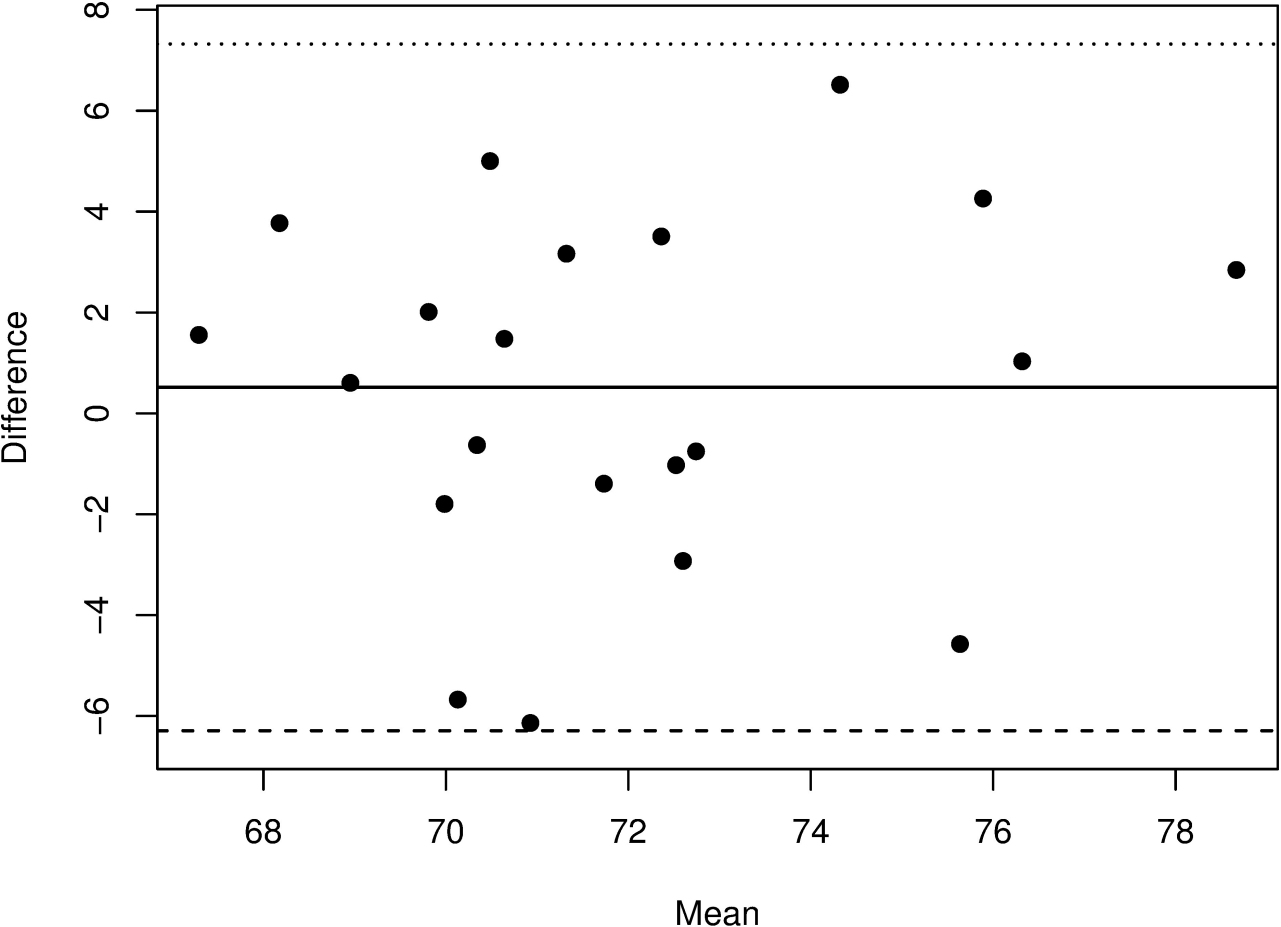
Bland-Altman plot comparing estimated and measured SO_2-transcranial_. Plot shows mean bias (solid line) and 95% limit of agreement (dashed lines).

The estimated SO_2-transcranial_ based on the fixed arterial:venous ratio of 30:70 showed a CCC of 0.46 (95% confidence interval: 0.05 to 0.73) with the measured SO_2-transcranial_. Bias between measured and estimated SO_2-transcranial_ was 0.51 with a 95% confidence and 95% tolerance interval of -1.07 to 2.10 and -13.70 to 14.72 respectively. Bland-Altman analysis is shown in Fig 3.

**Fig 4:**
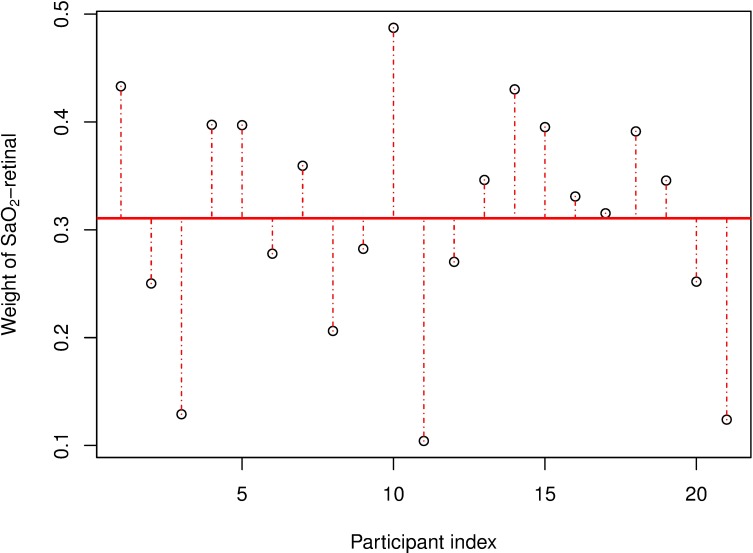
Relative weight of SaO_2-retinal_ to obtain measured SO_2-transcranial_. Plot showing individual arterial weights for each study participant (Eq 2). Average arterial weight is 0.31 (solid horizontal line) but inter-individual differences are large, ranging from 0.10 to 0.49.

The average relative weights of SaO_2-retinal_ and SvO_2-retinal_ to obtain the measured SO_2-transcranial_ were 0.31 ± 0.11 and 0.69 ± 0.11, respectively, with large inter-individual variability ranging from 10 to 49 for arterial weight, as summarized in Fig 4. The weighs for each individual are computed using Eq 2.

## Discussion

The human retina provides a unique view on the cerebral vasculature. Many studies have pointed out structural and functional homologies between the retinal and the cerebral circulation. [42–45] Structural parameters in the retinal vascular tree have been correlated with a variety of neurological diseases. [45,52,53] The added value of functional retinal vascular parameters has been investigated less extensively. Denninghoff et al demonstrated in a swine model that SvO_2-retinal_ correlates well with mixed venous oxygen saturation. [54] Mixed venous oxygen saturation is a valuable tool in the management of critically ill patients as a surrogate for cardiac output. In contrast to mixed venous oxygen saturation measured by a pulmonary artery catheter, retinal venous oxygen saturation can be measured non-invasively. This is the first study to investigate the correlation between retinal vessel oxygen saturation measured non-invasively by spectrophotometric oximetry, and cerebral oxygen saturation measured by NIRS.

First, this study shows that both SaO_2-retinal_ and SvO_2-retinal,_ measured by retinal oximetry, are positively correlated with SO_2-transcranial_, measured by NIRS. The analysis per vessel type revealed a significant correlation between measured and estimated SO_2-transcranial_. Where previous studies have demonstrated a significant correlation between SaO_2-retinal_ and SO_2-pulse_, our study could not reproduce this finding. [55] Of interest is the relative low retinal arterial saturation compared to SO_2-pulse_, which has been reported also in previous studies and is thought to result from a counter-current effect happening in the narrow space between the central retinal artery and vein inside the optic nerve. [56] SvO_2-retinal_ showed a borderline significance with estimated SvO_2-transcranial_. The above mentioned findings strengthen the hypothesis that the structural similarities between retinal and cerebral circulation also extend to a functional level. Apart from a common embryonic origin, the retinal and cerebral circulation have a common vascularization pattern during development, a non-fenestrated endothelial barrier, are covered by pericytes and glial cells and share the same control mechanisms. [43,45,57] Notable differences between both circulations include the lack of anastomoses and the distinct vascular branching pattern of the retinal circulation. [58] Local autoregulation also shows different responses between the cerebral and the retinal circulation with the retinal circulation being less reactive to hypercapnia and more reactive to hyperoxia. [59, 60] These differences in vascular reactivity should be taken into consideration when extrapolating cerebral oxygen saturation from retinal measurements.

Second, we found that the average relative partition of SaO_2-retinal_ and SvO_2-retinal_ to obtain the measured SO_2-transcranial_ closely matches the established arterial:venous ratio of 30:70 calibration of FORE-SIGHT^®^ NIRS technology. [18,20] Since the arteries supplying the retina and the frontal cortex are both direct branches of the internal carotid artery, it is reasonable to assume that the differences in arterial oxygen saturation between both circulations are small. However, the relative weight of SaO_2-retinal_ and SvO_2-retinal_ to SO_2-transcranial_ showed large inter-individual variation ranging from 10% to 49% for the arterial and from 51% to 90% for the venous contribution. These variations are in line with studies that investigated the relative contribution of arterial and venous cerebral oxygen saturation to SO_2-transcranial_. [16,20] The relatively low CCC between measured SO_2-transcranial_ and estimated SO_2-transcranial_ could therefore imply that retinal oximetry, being able to measure arterial and venous saturation separately, measures some additional information that is averaged out by NIRS.

Third, and of particular interest, is the negative correlation between retinal venous diameter and SO_2-transcranial_. As explained above, the retinal and cerebral circulation share similar vascular regulatory mechanisms. As such, a possible explanation for these findings could be that dilated retinal veins are indicative of cerebral venodilatation which would in turn result in a higher relative contribution of the venous compartment in SO_2-transcranial_. [43,44,61] Moreover, retinal venous diameter is known to increase in case of increased intracranial pressure. [62,63] Because of the known influence of vessel diameter on retinal oximetry measurements, a correction for vessel diameter is automatically applied by the provided software. [64, 65] Combined with the fact that retinal oximetry has the advantage of allowing to separately measure arterial and venous oxygen saturation, retinal oximetry is expected to be less influenced by vessel diameter than NIRS.

Taken together, we believe these findings provide proof of concept for the potential added value of retinal oximetry in assessing cerebral oxygen saturation. The ability of retinal oximetry to easily measure arterial and venous oxygen saturation separately, without "contamination" of superficial peripheral circulation may be especially relevant in situations where the relative contribution of arterial and venous saturation might deviate from the established arterial:venous ratio, for instance during extracorporeal circulation or in the post-resuscitation setting, when the accuracy of NIRS could be limited due to therapeutic hypothermia, vasopressor medication, alterations in (auto)regulation of the cerebral and superficial circulation,… [14,66]

Current retinal oximeters have several limitations that inhibit the use in the settings mentioned above. First, retinal oximetry provides a snapshot in time but not a continuous measurement. Next, the size of the current retinal oximeters limits its bedside usability. In addition, the price is steep. However, recently developed hyperspectral image sensors at the size of a regular color image sensor will likely provide an answer to both issues. [67] By replacing the color image sensor with a hyperspectral sensor, any portable funduscamera could be modified into a portable retinal oximeter. Provided that retinal oximetry could be performed at the bedside, this technology could become an interesting tool in the hemodynamic and cerebral assessment of critically ill patients and hence also contribute to a better understanding of NIRS.

The present study must be interpreted within the context of its potential limitations. First, the lack of exact measurements of the true cerebral saturation precludes firm conclusions about the correlation between retinal and cerebral oxygen saturation. While we believe several of our findings point towards this correlation, it remains to be investigated in future studies. Second, a major limitation of this study is the facts that measurements were performed only under normoxic conditions with an oxygen concentration of 21%. Future studies measuring desaturation data during graded hypoxia are warranted. Third, whereas SO_2-transcranial_ is measured continuously, retinal oximetry only provides a snapshot of the saturation in the retinal vessel at one particular moment. We tried to accommodate for this potential source of error by measuring the retinal oxygen saturation in steady state conditions after at least 20 minutes of adaptation in a seated position. However, this limitation might hamper future studies in non-steady state conditions. Last, we recognize the small sample size of the study. However, we believe this pilot study does provide a proof of concept for the relevant role of retinal oximetry in estimating cerebral oxygen saturation.

## Conclusion

In conclusion, this is the first study to describe the relation between retinal and cerebral oxygen saturation measured by retinal oximetry and NIRS. The ability of retinal oximetry to measure arterial and venous oxygen saturation separately without "contamination" of superficial peripheral circulation may have future applications in clinical practice. Further studies are warranted to investigate the exact relation of retinal oximetry with cerebral and systemic oxygen saturation.

## Supporting information

S1 File Raw data. Individual data points for all subjects.
(CSV)

S2 File Statistical analyses and figures. R script for all analyses and figures included in the paper.
(R) Click here for additional data file.
